# Parkin paves the path to antitumor immunity: Expanding Parkin’s role as a tumor suppressor

**DOI:** 10.1172/JCI185838

**Published:** 2024-11-15

**Authors:** Hyungsoo Kim, Ze’ev A. Ronai

**Affiliations:** Departments of Surgery and Biomedical Sciences, Translational Research Institute, Cedars Sinai Medical Center, Los Angeles, USA.

## Abstract

Parkin, a ring-between-ring-type E3 ubiquitin ligase, first shown to play a critical role in autosomal recessive juvenile Parkinsonism, has recently emerged as a key player in cancer biology. Parkin is now known to serve as a tumor suppressor, and its deregulation frequently promotes tumorigenesis. In this issue of the *JCI*, Perego et al. expand that role by showing that Parkin expression stimulated an interferon (IFN) response to modulate CD8^+^ T cell activity. These findings suggest that, in addition to directly inhibiting tumor progression, Parkin enhances antitumor immune responses, highlighting it as a promising therapeutic target for cancer treatment.

## Parkin’s roles in cancer

Encoded by the PRKN gene (also known as PARK2), Parkin, a causative gene for autosomal recessive juvenile Parkinsonism (ARJP), serves as a multifunctional E3 ligase (1, 2) that regulates activities as diverse as mitochondrial quality control (including mitophagy), cell cycle progression, cell death, the DNA damage response, genomic stability, cellular metabolism, and inflammation (3, 4). Parkin is frequently deregulated in cancer, and genetic alterations — such as mutations or deletions — and epigenetic modifications commonly reported in breast, lung, colorectal, and pancreatic cancers (3) often result in partial or total loss of Parkin function. These changes lead to unchecked cellular proliferation and resistance to apoptosis, genomic instability, increased cell motility, and metabolic reprogrammings — all hallmarks of cancers — and contribute to tumor growth, progression, and metastasis.

In Parkinson’s disease, impaired Parkin function promotes defects in the major mitochondrial quality control process known as mitophagy, an activity that prevents accumulation of damaged mitochondria. Mitochondrial injury releases damage-associated molecular patterns (DAMPs) that activate STING-mediated inflammation and, in the absence of mitophagy, these signals correlate with autoimmune phenotypes (5, 6). Perturbations in mitophagy due to Parkin deficiency also augment antiviral immune responses through ROS-mediated activation of the inflammasome, which can impair viral clearance (7). Finding the role of Parkin in regulating inflammation points to its crucial role in antitumor immunity.

## Parkin’s involvement in antitumor immunity

In this issue of the *JCI*, Perego and colleagues explored tumor-related functions and revealed that Parkin was epigenetically silenced (by promoter hypermethylation) in various cancers. Further, clinically approved demethylating therapies that promoted Parkin’s reexpression stimulated an IFN response, which is a critical component of innate immunity (8). Parkin’s E3 ligase activity was essential for this IFN response, which involved subcellular trafficking and the release of High Mobility Group Box 1 (HMGB1), an alarmin that triggers immune responses by activating cGAS-STING. The finding of a Parkin/HMGB1 axis is unique, as increasing mtDNA and ROS production via Parkin expression was not sufficient, but was necessary to activate IFN response. Parkin reexpression activated STAT1, a key transcription factor in the IFN response, while inhibiting NF-κB gene expression and STAT3 phosphorylation, key players in inflammation (9). Interestingly, NF-κB and STAT3 activities are often associated with tumor growth and cell survival (9, 10), indicating that Parkin may suppress both protumorigenic pathways.

Perego and authors also demonstrated that Parkin activation of IFN signaling had downstream effects on the immune system; notably, Parkin-mediated signaling led to paracrine activation of CD8^+^ T cells (8). Those activated CD8^+^ T cells exhibited reduced expression of the immune inhibitory receptors TIM3 and LAG3, which are often upregulated in the tumor microenvironment to dampen immune responses (11, 12). By contrast, activated T cells expressed higher levels of TCF1, a factor associated with stem cell–like properties and self renewal, sustaining antitumor activity (13). Consequently, Parkin activity increased the capacity of exhausted effector CD8^+^ T cells to become reinvigorated into a cytotoxic state that better responded to immune checkpoint inhibitors (ICI) (14, 15).

## Conclusions and clinical implications

**Figure 1:**
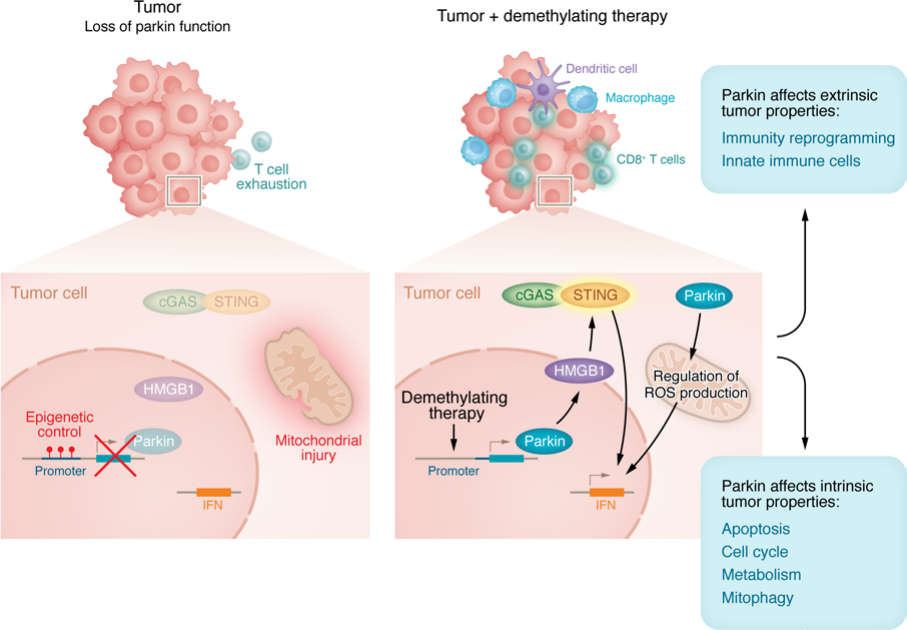
Parkin influences tumor immunity through its intrinsic and extrinsic functions. Parkin is genetically and epigenetically deregulated in cancer. Loss of Parkin results in mitochondrial injury and reduced immune function, including T cell exhaustion. Activity of Parkin E3 ligase within tumor cells after restoration by demethylating therapy alters subcellular trafficking and the release of HMGB1, which activates the GAS/STING pathway. The latter, in turn, activates the IFN pathway, which leads to paracrine activation of CD8^+^ T cells and innate immune cells, ultimately stimulating antitumor immunity.

Overall, these findings suggest that Parkin functions as a dual-mode tumor suppressor. On the one hand, it directly inhibits tumor-intrinsic activities to counteract oncogenic metabolism and changes in cell motility required for tumor progression and metastasis, respectively (3). On the other hand, Parkin indirectly enhances extrinsic antitumor immunity by reinvigorating CD8^+^ T cells within the tumor microenvironment (Figure 1). These findings strongly suggest that Parkin could serve as a therapeutic target in cancer treatment, especially in strategies aimed at reactivating silenced tumor suppressor genes to boost intrinsic and extrinsic immune-mediated tumor suppression.

The findings of Perego and colleagues also raise several questions for future studies. Among those are: (a) How does Parkin E3 ligase activity regulate the HMGB1-cGAS/STING axis to activate IFN signaling and innate immunity? Specifically, how does HMGB1 that emerged as a target of Parkin (4) activate cGAS/STING? (b) Given that diverse cellular signals deregulate Parkin in cancer cells, how do other types of deregulation (such as posttranscriptional modifications) impact IFN signaling and innate immunity? (c) How can Parkin’s antitumor function be exploited to develop improved therapeutic strategies, including combination therapies with ICI regimens? (d) Which cohorts of patients with cancer exhibit Parkin deregulation, and are they amenable to related therapies?

In all, Perego et al. (8) provides compelling evidence that Parkin serves as a critical regulator of innate immunity in the context of cancer and highlights its potential as a therapeutic target, which is expected to enhance antitumor responses. Epigenetic regulation of Parkin and its impact on both tumor cells and the immune microenvironment underscore the complexity of tumor biology and how innovative cancer therapies will be required to target these mechanisms.

## Version 1 11/15/2024

Electronic publication
